# Medical Gaslighting in Urban Healthcare: A Bioecological Framework for Health Equity and Participatory System Transformation

**DOI:** 10.3390/healthcare14193244

**Published:** 2026-10-01

**Authors:** Anthony D. Slonim, Mustafa Naveed, Kryss Shane

**Affiliations:** 1 Departments of Internal Medicine, Pediatrics, Health Systems and Interprofessional Sciences, Virginia Tech Carilion School of Medicine, Roanoke, VA 24014, USA; 2 Department of Internal Medicine, Virginia Tech Carilion School of Medicine, Roanoke, VA 24014, USA; mnaveed@carilionclinic.org; 3 Department of Social Work, National University, San Diego, CA 92123, USA; kryss.shane@gmail.com; 4 School of Education, Endicott College, Beverly, MA 01915, USA

**Keywords:** medical gaslighting, urban health, health equity, epistemic injustice, bioecological theory, patient-centered care, participatory research, healthcare systems, trust, diagnostic overshadowing, care fragmentation

## Abstract

Medical gaslighting describes patient experiences in which symptoms, concerns, or interpretations of illness are minimized, dismissed, or insufficiently recognized within healthcare encounters. While the term initially emerged from patient and community narratives, recent empirical and conceptual studies (2019–2024) have further documented patterns of diagnostic overshadowing, credibility deficits, and structural contributors to patient invalidation. These findings indicate that interpersonal dismissal often reflects broader organizational and systemic dynamics. This paper proposes a bioecological framework for understanding medical gaslighting as a multi-level phenomenon within urban healthcare. Drawing on Bronfenbrenner’s bio-ecological theory, Fricker’s concept of epistemic injustice, Lipsky’s theory of street-level bureaucracy, and participatory traditions advanced by Dewey, Freire, and community-based participatory research, the framework examines how experiences of invalidation arise through interactions among patients, clinicians, healthcare organizations, policies, and communities. Participatory approaches position patients and communities as partners in defining problems, generating knowledge, and designing solutions. This framework conceptually advances participatory knowledge production as a mechanism for organizational learning and system transformation. Addressing medical gaslighting requires more than improving communication; it requires recognizing lived experience as legitimate knowledge and redesigning healthcare systems to support partnership, continuity, and learning from the patients and communities they serve. Because this model has not yet been empirically validated, its components should be interpreted as theoretical propositions intended to guide future research. By connecting individual experiences to system-level transformation, healthcare organizations can strengthen equity, trust and human-centered care.

## 1. Introduction

Urban healthcare systems are designed to diagnose illness, reduce suffering and improve population health. Although these systems provide advanced clinical expertise and serve as essential resources for diverse communities, many patients describe healthcare encounters where their symptoms, concerns, or lived experiences are insufficiently recognized. The term *medical gaslighting* has emerged from patient and community discourse to describe situations where individuals feel dismissed, disbelieved, or are repeatedly required to establish the legitimacy of their symptoms before receiving appropriate evaluation. Recent empirical studies have documented similar patterns of diagnostic overshadowing, credibility deficits, and symptom minimization across diverse clinical contexts [1,2,3].

The emergence of this terminology is significant because it reflects patients’ attempts to describe experiences that have not been adequately captured by traditional medical language. They report recurring patterns in which symptoms are prematurely attributed to psychological causes, diagnostic possibilities are narrowed without sufficient exploration, or prior assumptions influence subsequent care decisions. The growing use of the term in both public discourse and academic literature suggests broader concerns regarding recognition, authority, equity, and trust within healthcare relationships [4,5].

Urban healthcare environments are particularly relevant for examining these experiences because they combine sophisticated medical resources with complex organizational structures. Patients navigate interconnected networks of primary care practices, specialty clinics, emergency departments, hospitals, insurers, and community organizations. For individuals with chronic illness, diagnostic uncertainty, or multiple conditions, this complexity may create fragmented care requiring repeated explanations across disconnected settings. Urban systems also disproportionately serve populations experiencing structural inequities, making them uniquely susceptible to multi-level forms of invalidation [6,7].

As continuity becomes more difficult to maintain, the responsibility for integrating care frequently shifts from healthcare systems to patients and families. Individuals must communicate prior evaluations, reconcile conflicting recommendations, navigate referrals, advocate for additional investigations, and preserve a coherent narrative of their illness. These demands place patients in the position of managing not only their health conditions, but also the information systems intended to support their care. Research on care fragmentation demonstrates that these burdens are especially pronounced in urban environments, where organizational interfaces are numerous and often poorly communicated [8].

Medical gaslighting is therefore inadequately explained as solely an interpersonal communication problem. Clinical encounters occur within organizational and structural environments that influence how clinicians interpret information, manage uncertainty, allocate attention, and determine which forms of knowledge are incorporated into decision-making. Empirical studies show that time pressure, documentation requirements, and productivity expectations can constrain clinicians’ ability to fully explore patient concerns, contributing to patterns of dismissal that are not solely interpersonal [9,10].

Each healthcare encounter involves the interaction of multiple forms of knowledge. Clinicians contribute biomedical expertise, diagnostic reasoning, and professional experience. Patients contribute experiential knowledge developed through living with illness, recognizing symptom patterns, understanding functional limitations, and interpreting the disease within the context of daily life. High-quality healthcare depends on integrating these complementary sources of knowledge.

Healthcare systems, however, have historically prioritized certain forms of knowledge over others. Fricker’s concept of epistemic injustice provides an important framework for understanding how individuals may experience diminished credibility as knowers when their testimony is discounted or interpreted through assumptions unrelated to their actual experiences [11]. Within healthcare, this may occur when symptoms do not align with expected disease patterns, when prior diagnoses constrain interpretation of new concerns, or when social assumptions influence clinical judgment. Recent work has extended epistemic injustice theory into clinical settings, demonstrating how credibility deficits shape diagnostic reasoning and patient–provider communication [1,12].

Evidence also demonstrates that patient experiences and symptom reports may be interpreted differently across social groups. Hoffman et al. demonstrated that racial bias and inaccurate beliefs about biological differences can influence pain assessment and treatment recommendations, illustrating how credibility and interpretation are shaped by broader social contexts [13]. Additional studies have shown similar disparities in diagnostic delay, symptom dismissal, and treatment recommendations across racial, gender, and socioeconomic groups [4,7].

Although epistemic injustice helps explain how patient knowledge may be discounted, it does not fully explain why these patterns recur across healthcare systems. Clinicians practice within environments characterized by time limitations, documentation requirements, productivity expectations, resource constraints, and competing priorities. Lipsky’s theory of street-level bureaucracy explains how front-line professionals adapt decision-making when institutional demands exceed available resources [14]. This framework has been applied to healthcare to explain how clinicians rely on heuristics, root sanitization, and narrow diagnostic pathways under pressure [15].

Bronfenbrenner’s bioecological theory provides an organizing framework for examining these interconnected influences [16]. Rather than viewing healthcare encounters as isolated interactions, this framework recognizes that experiences emerge through relationships among individuals, organizations, institutions, policies, and historical contexts.

**Figure 1:**
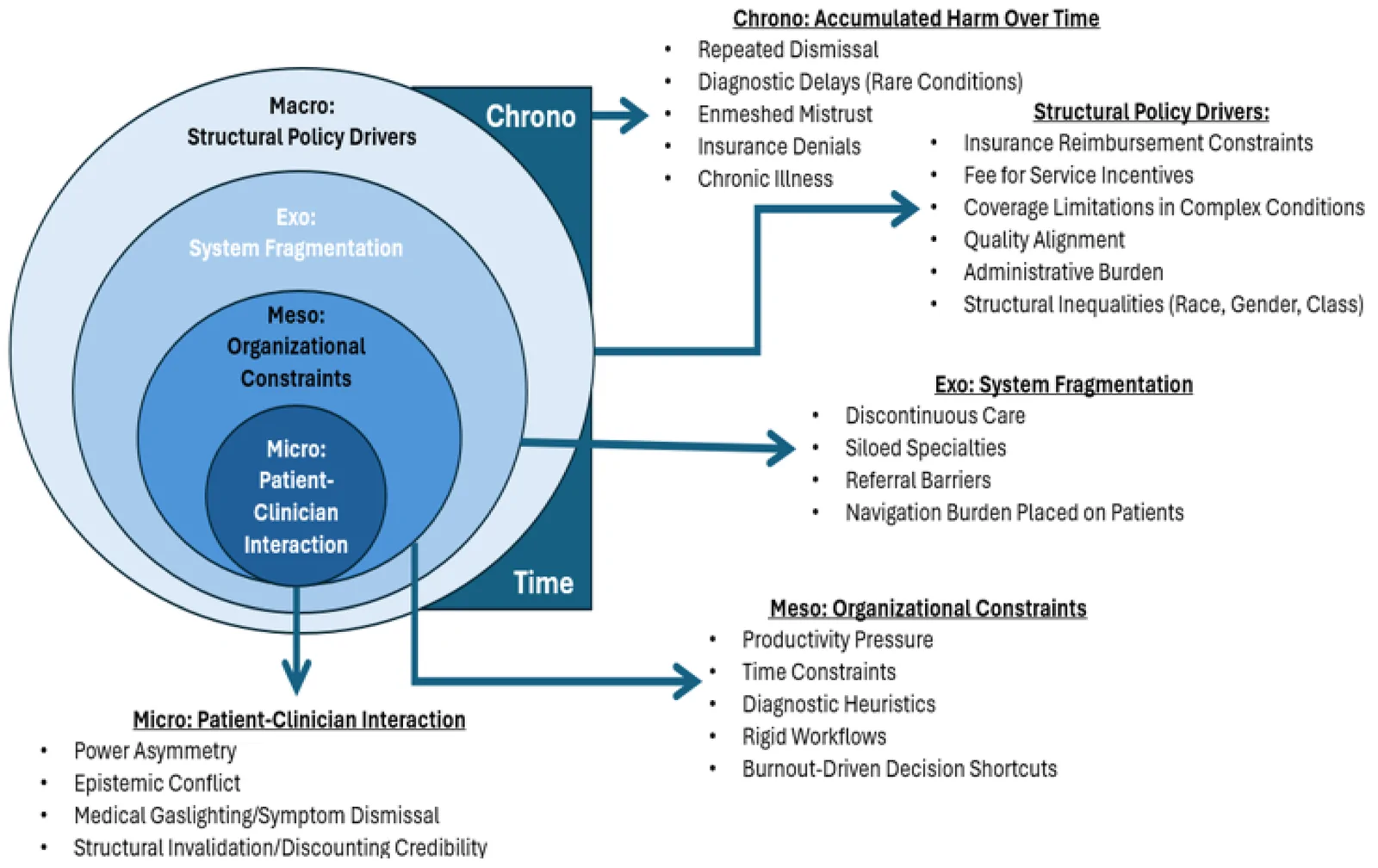
Bioecological framework for medical gaslighting in urban healthcare. Conceptual model applying Bronfenbrenner’s bioecological framework to medical gaslighting. The model demonstrates how experiences of invalidation emerge through interactions across nested healthcare environments. The microsystem represents patient–clinician interactions and recognition of patient knowledge. The mesosystem represents healthcare organizations and operational constraints. The exosystem represents health system structure influencing continuity and access. The macrosystem represents policy, financing, and structural inequities. The chronosystem represents accumulated experiences over time and their effects on trust, engagement and healthcare.

Applied to medical gaslighting, the bioecological framework suggests that experiences of invalidation emerge through interactions across multiple levels of healthcare systems (Figure 1). At the microsystem level, patient–clinician interactions shape whether knowledge is recognized. The mesosystem encompasses organizational conditions that shape clinicians’ ability to provide relationship-centered care. The exosystem includes health infrastructure, referral systems, insurance processes, and information systems that influence continuity. The macrosystem reflects policies, financing structures and social inequities that shape healthcare priorities. The chronosystem recognizes how repeated experiences accumulate over time and influence trust, engagement and future healthcare behaviors (Figure 1). This multi-level framing highlights the need for conceptual models that connect interpersonal experiences to organizational learning and system transformation, an area not yet fully addressed in the existing literature.

This framework does not suggest that every disagreement between patients and clinicians represents medical gaslighting. Medicine requires interpretation, uncertainty and professional judgment. The concern arises when disagreement occurs within patterns of dismissal, when patient knowledge is systematically discounted [17] or when institutional structures repeatedly prevent meaningful recognition [1,2,12].

Understanding these experiences requires moving beyond individual-level interventions. Communication training remains important, but communication alone cannot resolve organizational conditions that constrain time, continuity and partnership. Healthcare systems must also examine how they generate knowledge, how they respond to patient experiences, and how communities participate in defining solutions [18].

Participatory approaches provide an important pathway for transforming these systems. Dewey’s theory of experiential learning emphasizes that knowledge emerges through reflection, inquiry, and action based on experience [19]. Freire’s work on dialogue and shared authority argues that meaningful transformation requires relationships in which individuals participate as contributors to knowledge rather than passive recipients of expertise [20]. Community-based participatory research extends these principles by demonstrating how equitable partnerships between institutions and communities can generate relevant knowledge and sustainable solutions [21].

These principles align with the concept of learning health systems, which emphasize continuous improvement through the integration of evidence, operational data, and patient experience [22]. For urban healthcare systems, this approach is particularly important because communities experiencing health inequities possess essential knowledge regarding barriers, institutional relationships, and unmet needs that may not be visible through traditional healthcare metrics [6,8].

Accordingly, this paper proposes that medical gaslighting is an important feature of urban healthcare systems rather than solely an interpersonal communication failure. Using a bioecological framework, we examine how experiences of invalidation develop across healthcare environments and identify participatory knowledge production as a mechanism for organizational learning, health equity, and system transformation. Because this model has not yet undergone empirical validation, its proposed mechanisms should be interpreted as theoretical contributions intended to guide future research.

## 2. Conceptual Development and Methodology

This paper uses a narrative and conceptual synthesis approach rather than a systematic review methodology. The purpose was to integrate literature addressing medical gaslighting and related experiences of symptom dismissal, diagnostic overshadowing, credibility deficits, epistemic injustice, healthcare fragmentation, organizational constraints, and patient and community participation. Relevant literature was identified through iterative searches of the scholarly literature and examination of references from conceptual and empirical publications. The literature search was intentionally broad because the terminology used to describe medical gaslighting is evolving and has not yet been standardized across clinical, social science, and health services research.

Literature was selected based on its relevance to one or more of the following domains: (1). Patient experiences of dismissal, invalidation, or reduced credibility in healthcare. (2). Diagnostic reasoning and diagnostic overshadowing. (3). Organizational and structural factors influencing clinical encounters. (4). Healthcare fragmentation, continuity, and trust. (5). Participatory approaches to knowledge production, organizational learning, and healthcare improvement. Particular attention was given to recent empirical and conceptual work while retaining foundational theoretical literature necessary to establish the conceptual framework.

The theoretical frameworks were selected according to their ability to explain complementary components of the phenomenon rather than because they represent competing explanations. Bronfenbrenner’s bioecological theory was selected as the overarching organizing framework because it provides a structure for examining interactions across individual, relational, organizational, system, policy, and temporal levels. Fricker’s theory of epistemic injustice was used to explain how patient knowledge and credibility may be discounted within clinical encounters. Lipsky’s theory of street-level bureaucracy was incorporated to examine how frontline clinicians’ decisions may be shaped by organizational constraints, competing demands, and limited resources. Dewey’s theory of experiential learning, Freire’s work on dialogue and shared authority, and community-based participatory research were incorporated to provide a conceptual basis for transforming patient and community experience into shared knowledge and organizational learning.

The proposed model was developed by synthesizing these complementary perspectives and mapping their concepts onto the levels of the bioecological framework. The resulting model links experiences at the clinical encounter level with organizational processes, health system structures, broader policy and social conditions, and accumulated experiences over time. A second component of the model extends this framework by conceptualizing participatory knowledge production as a potential mechanism through which patient and community experience can inform organizational learning and system change. The resulting framework was refined through iterative comparison of the theoretical concepts with themes identified in the reviewed literature and is presented as a set of conceptual propositions rather than as an empirically validated causal model. Because this is a conceptual or narrative review, no formal systematic review protocol, meta-analysis, or quantitative synthesis was undertaken. The purpose of the literature synthesis was to develop and clarify a theoretically grounded framework that can generate testable propositions for future empirical research.

## 3. Medical Gaslighting as an Emergent Bioecological Phenomenon in Urban Healthcare

The term medical gaslighting has become increasingly prominent because it provides language for a specific type of healthcare experience: the perception that a patient’s symptoms, concerns, or interpretations are repeatedly minimized, excluded from clinical reasoning or insufficiently recognized. The term emerged primarily through patient narratives and public discourse rather than through traditional academic medicine. Its emergence is significant because it reflects an attempt by patients and communities to describe experiences that existing healthcare terminology did not adequately capture. Recent qualitative studies have further documented these patterns across chronic illness, women’s health, and racialized patient populations [2,3,4,7].

A systems-oriented analysis recognizes two principles simultaneously. First, patients’ reports of dismissal represent meaningful information about healthcare systems’ performance. Repeated experiences of not being believed, not being understood, or not having concerns integrated into decision-making may indicate failure in healthcare processes, relationships, or institutional learning. Second, not all clinical disagreement represents invalidation. The central question is not whether disagreement occurs, but whether disagreement occurs within a relationship that recognizes the patient as a credible contributor to understanding their own health. This distinction aligns with emerging empirical work on credibility deficits and epistemic exclusion in clinical encounters [1,12].

This distinction is especially important in urban healthcare environments, where complex medical and social needs intersect with highly specialized, but often fragmented systems of care. Patients interact with primary care, specialty services, urgent care facilities, emergency departments, community organizations and insurers each with their own priorities, workflows, and information systems. These organizational interfaces influence whether patient narratives remain coherent across time and settings. Urban systems, characterized by high patient volume and structural inequities, may amplify these challenges [6,8].

For patients with persistent symptoms, chronic illness, or diagnostic uncertainty, fragmentation may require repeated reconstruction of medical histories, reconciliation of conflicting recommendations, and continued advocacy for additional evaluation. Over time, these experiences shape perceptions about whether healthcare systems are responsive, trustworthy, and capable of recognizing individual needs. Studies of care fragmentation show that these burdens disproportionately affect patients with multimorbidity and those navigating complex urban systems [8].

Urban health disparities further influence these experiences. Social determinants of health, including transportation barriers, housing instability, economic insecurity, language differences and unequal exposure to discrimination affect both access to healthcare and the context in which healthcare interactions occur. Social determinants contribute to health inequities by shaping the conditions in which people live, work and seek care [13,14]. Structural racism and historical patterns of exclusion also influence relationships between communities and healthcare institutions [15]. These influences are particularly relevant in urban environments, where diverse populations often experience concentrated social and healthcare challenges [16]. These structural factors shape not only health outcomes but also the interpretive environment in which patient concerns are evaluated.

These structural conditions do not determine individual clinical encounters, nor do they eliminate individual responsibility. Rather, they establish environments in which those encounters occur. Farmer’s analysis of structural violence demonstrates how social and institutional arrangements can produce unequal health experience even when individuals may not intend harm [17]. This perspective underscores that medical gaslighting may emerge from systemic configurations rather than solely interpersonal failures.

The clinical encounter represents the point at which recognition or dismissal becomes visible, but the forces shaping that interaction extend beyond the examination room. A bioecological framework therefore provides a mechanism for understanding how individual experiences emerge from interactions among patients, clinicians, organizations, policies, and communities.

## 4. A Bioecological Framework for Medical Gaslighting

Bronfenbrenner’s bioecological theory proposes that human experiences develop through interactions among multiple nested environmental systems rather than through isolated individual events [16]. Applied to healthcare, this framework provides a structure for examining how experiences described as medical gaslighting may emerge across interconnected levels. This multi-level perspective fills a gap in the existing literature by linking epistemic injustice to organizational learning and system-level dynamics.

This framework does not identify a single cause of medical gaslighting. Instead, it demonstrates how multiple factors may interact to create environments where patient knowledge is recognized, discounted, or transformed into institutional learning (Figure 1).

### 4.1. Microsystem: Patient–Clinician Interaction and Epistemic Recognition

The microsystem represents the immediate environment where patients and clinicians interact. It is the level at which experiences of recognition or invalidation become most visible.

Effective care requires integration of two complementary forms of expertise. Clinicians contribute biomedical knowledge, diagnostic reasoning, and professional experience. Patients contribute experiential knowledge developed through their bodies, symptoms, daily functioning and illness trajectories. When these knowledge sources are integrated, the inherent asymmetry of clinical authority can be balanced by partnership.

Fricker’s concept of epistemic injustice provides an important theoretical foundation for understanding situations in which patients experience reduced credibility as contributors to knowledge [11]. Within healthcare, epistemic injustice may occur when symptoms are interpreted primarily through assumptions rather than through exploration, when patients are viewed as unreliable because their symptoms are complex, or when social stereotypes influence clinical judgement. Empirical studies have shown that credibility deficits can shape diagnostic pathways, delay evaluation, and reduce the likelihood that patient concerns are incorporated into decision-making [1,2].

Some examples include symptoms being prematurely attributed to stress or psychological causes, concerns being discounted because objective findings are limited, previous diagnoses influencing interpretation of new symptoms, or patients needing repeated advocacy before additional evaluation occurs. These experiences may be particularly relevant for individuals with complex, chronic, or poorly understood conditions.

Recognizing epistemic injustice does not require replacing clinical expertise with patient interpretation. Rather, it requires understanding that clinical expertise functions most effectively when combined with patients as knowledgeable participants in their own care.

The central microsystem question is: *Does the clinical interaction preserve the patient’s role as a legitimate participant in knowledge creation?*

When patients repeatedly experience dismissal, consequences may extend beyond dissatisfaction. Reduced trust, delays in seeking care, delayed diagnosis, decreased disclosure and disengagement with the health system may occur over time [18,19]. These downstream effects highlight the importance of epistemic recognition as a clinical competency.

### 4.2. Mesosystem: Healthcare Organizations and the Constraints of Care Delivery

The mesosystem represents the organizational environments connecting patients and clinicians including care teams, clinics, hospitals and health networks.

Although medical gaslighting becomes visible during individual encounters, those encounters are shaped by organizational conditions. Healthcare professionals practice within systems that determine appointment length, documentation expectations, staffing models, referral processes, and performance requirements.

Lipsky’s theory of street-level bureaucracy explains how professionals working within complex systems adapt their decisions when institutional expectations exceed available resources [14]. Healthcare clinicians frequently balance competing demands: providing individualized care while maintaining productivity expectations, exploring uncertainty while maintaining efficiency, and developing relationships while completing administrative responsibilities.

Research examining physician time allocation demonstrates the increased complexity of clinical work and the competing demands placed on clinicians during ambulatory care [20]. These pressures can influence how much time is available for listening, exploration, and relationship building.

Under these conditions, clinicians may rely on established diagnostic pathways, prioritize immediate concerns, or limit exploration of unresolved symptoms. These responses may represent adaptations to organizational constraints rather than intentional dismissal. However, patients experiencing these interactions may perceive them as evidence that their concerns are not valued.

Care coordination presents another organizational challenge. Fragmented systems require patients to navigate multiple providers and services, often without adequate support. Bodenheimer described care coordination as a persistent challenge within complex healthcare environments, particularly for patients requiring services across multiple settings [23].

The central mesosystem question becomes: *Do healthcare organizations create conditions that support recognition, continuity, and relationship-centered care?*

Organizational interventions include redesigning workflows, strengthening team-based care, reducing unnecessary administrative burden, and developing measures that evaluate trust, continuity and patient experience.

### 4.3. Exosystem: Health System Fragmentation and Continuity Failures

The exosystem includes healthcare structures that influence patient experiences without direct interaction. These include referral networks, insurance processes, electronic health records, and relationships among healthcare organizations.

For patients with complex conditions, fragmentation creates substantial burdens. Healthcare systems often excel at transferring discrete information such as laboratory results, imaging findings, medication lists, and diagnoses. However, complex illness requires continuity of meaning: understanding how symptoms developed, what has already been attempted, what remains uncertain, and how illness affects daily life.

When narrative continuity is lost, patients may feel required to repeatedly establish the legitimacy of their concerns. Administrative barriers, referral delays, insurance authorization requirements, and scheduling limitations may become experienced as signals that their needs are not prioritized.

Health Services Research emphasizes that healthcare experiences are shaped not only by clinical outcomes, but also by the processes through which patients interact with healthcare organizations [24]. A system that efficiently transfers data but fails to preserve patient narratives may unintentionally create conditions in which patients experience repeated invalidation.

The exosystem question becomes: *Does the healthcare system preserve patient knowledge across transitions of care?*

Solutions include integrated information systems, patient navigation, improved coordination, and organizational processes designed to maintain continuity across settings.

### 4.4. Macrosystem: Policy, Financing, and Structural Inequality

The macrosystem represents broader social, political, and economic environments influencing healthcare delivery.

Healthcare organizations operate within financing systems, regulatory environments, and historical patterns of inequality that shape institutional priorities. Although healthcare systems increasingly emphasize equity and patient-centered care, organizational incentives may not always support the time, continuity, and relationship-building required for these goals.

Payment structures frequently prioritize measurable productivity and episodic encounters, while complex patients often require longitudinal relationships, coordination, and uncertainty management. These tensions may disproportionately influence both health outcomes and interactions with healthcare institutions [13,14,15]. The effects of these structural forces become visible at the individual level: a restrictive insurance policy may be experienced as disbelief, a delayed referral as abandonment, and a brief appointment as dismissal.

The macrosystem question becomes: *Do healthcare policies and financing structures support recognition, continuity, and equitable relationships between patients and healthcare systems?*

Addressing these issues requires alignment of healthcare incentives with relationship-centered care, including financing approaches that support complexity, continuity, equity-focused accountability, and community partnerships.

### 4.5. Chronosystem: Time, Accumulated Experience, and the Development of Distrust

The chronosystem introduces time as a central component of medical gaslighting experiences. Patients rarely describe invalidation as a single isolated interaction. Instead, they often describe patterns developing across months or years through encounters with multiple clinicians and organizations.

Repeated experiences of not being heard may influence future healthcare behaviors. Patients may delay seeking care, reduce disclosure of symptoms, avoid healthcare settings, anticipate skepticism, or seek alternative sources of information outside of traditional systems. These responses should be understood within the context of accumulated experiences rather than as isolated individual choices.

Trust develops through repeated interactions between institutions and communities. Historical experiences of inequity influence how healthcare systems are perceived and how communities interpret current encounters.

The chronosystem question becomes: *How do repeated healthcare experiences shape future relationships between individuals, communities, and healthcare systems?*

Addressing medical gaslighting therefore requires attention not only to individual encounters but also to long-term institutional relationships with the populations that healthcare systems serve.

### 4.6. Integrating the Bioecological Framework

The bioecological framework demonstrates that medical gaslighting is not produced at a single level of healthcare delivery. Instead, it emerges through interactions among multiple systems.

At the microsystem level, patients experience whether their knowledge is recognized within clinical encounters. At the mesosystem level, organizations determine whether clinicians have the time and resources required for relationship-centered care. At the exosystem level, fragmented structures influence continuity and the preservation of patient narratives. At the macrosystem level, policies and financing structures establish institutional priorities. The chronosystem demonstrates how accumulated experiences shape trust, engagement, and future healthcare behaviors.

This framework reframes medical gaslighting from an isolated interpersonal problem into a signal of how healthcare systems manage knowledge, uncertainty, and relationships. The central question shifts from ‘Why did a clinician fail to listen?’ to ‘What conditions within healthcare systems make recognition, listening, and partnership difficult to sustain?’ This shift is important because meaningful interventions must address both the encounter and the systems surrounding it. Because this model has not yet been empirically validated, these mechanisms should be interpreted as theoretical propositions intended to guide future research.

## 5. Participatory Knowledge Production as a System Repair Mechanism

The bioecological framework demonstrates that medical gaslighting emerges through interactions among multiple levels of healthcare systems. Identifying how invalidation develops, however, is only the first step. If these experiences reflect failures of recognition, institutional learning, and knowledge integration, then meaningful solutions must address how healthcare systems generate value, and apply knowledge. Recent scholarship on organizational learning and patient reported experience underscores that systems often fail to incorporate experiential knowledge into improvement processes [18,22].

A central argument of this framework is that medical gaslighting represents not only a communication challenge, but also a problem of knowledge production. Healthcare systems continuously determine whose experiences are considered credible, how problems are defined, and which forms of evidence influence improvement efforts. When patient knowledge is repeatedly marginalized, healthcare systems lose meaningful knowledge about how their systems function. This dynamic parallels findings in epistemic injustice research, which show that excluding patient testimony reduces the accuracy and responsiveness of clinical decision-making [1,12].

**Figure 2:**
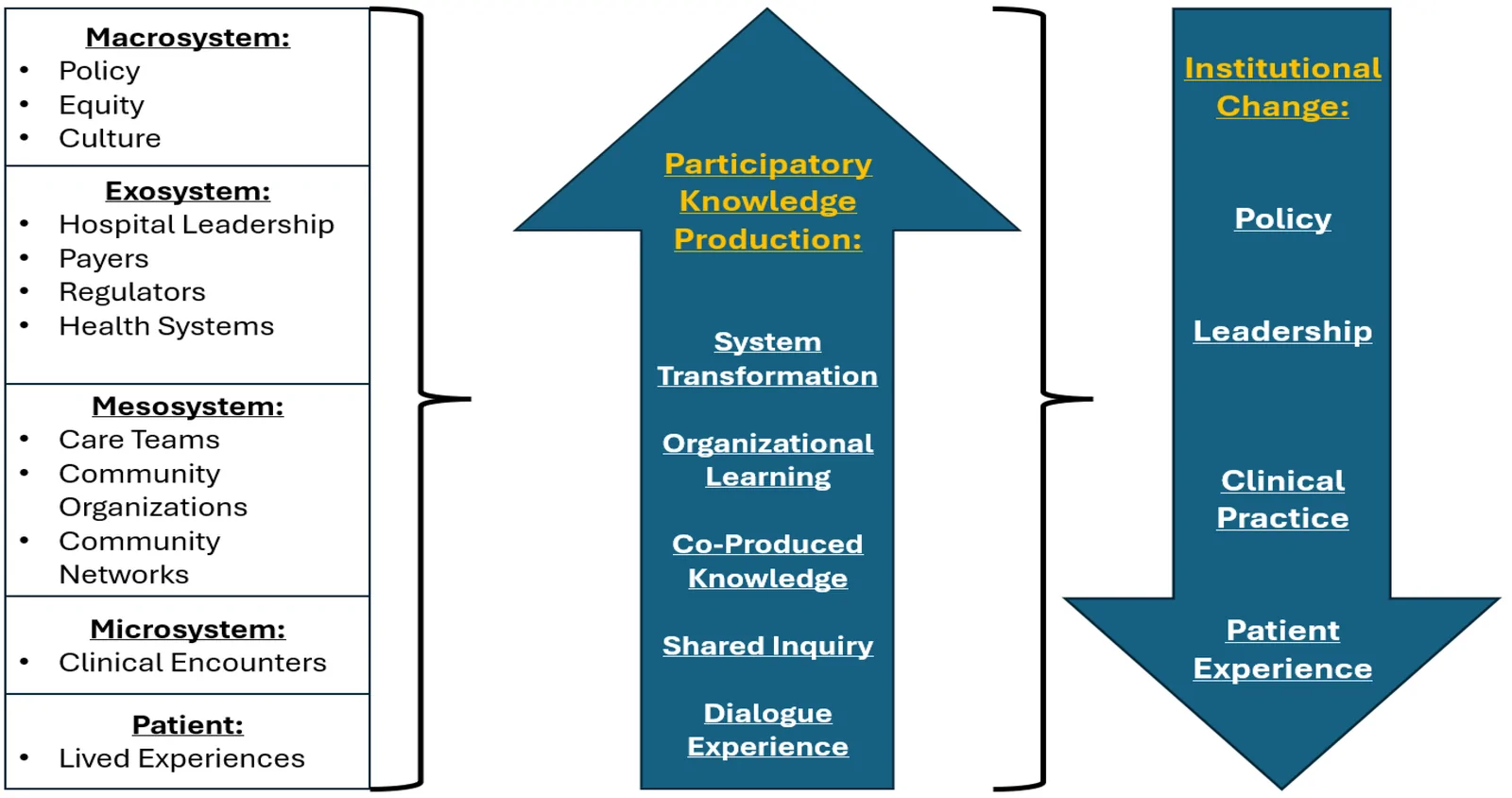
Participatory knowledge production as a mechanism for healthcare system learning. Conceptual model illustrating how patient and community experiences are transformed into organizational knowledge through participatory processes. Drawing on Dewey’s theory of experiential learning, Freire’s dialogical approach, and principles of community-based participatory research, the model demonstrates movement from lived experience to shared inquiry, co-produced knowledge, organizational learning, and system transformation. Patients and communities function not only as sources of feedback, but as partners in defining problems, interpreting findings, designing interventions, and evaluating outcomes.

Participatory approaches provide a pathway for transformation by positioning patients and communities as partners in generating knowledge rather than passive recipients of healthcare services. This approach shifts patient experience from a satisfaction metric to a source of organizational change (Figure 2). Participatory methods, including co-design, community-based participatory research, and experience-based co-production, have demonstrated effectiveness in generating actionable insights and improving system responsiveness [21,25].

## 6. Experience as a Foundation for Institutional Learning

John Dewey’s theory of experience provides a foundational rationale for participatory healthcare improvement [19]. Dewey argued that meaningful learning occurs through cycles of experience, reflection, inquiry and action. Experience alone does not automatically produce knowledge; institutions must actively examine what experiences reveal and determine how those lessons should influence future action. This perspective aligns with contemporary learning health systems frameworks emphasizing continuous feedback loops and iterative improvement [22].

Applied to healthcare, this perspective suggests that patient experiences are not merely indicators of satisfaction but sources of information about system performance. When patients describe repeated dismissal of symptoms, difficulty navigating referrals, inability to maintain continuity, or exclusion from decision-making, they provide insight into healthcare processes from the perspective of those who experience them directly. A Deweyan approach changes the institutional question “What does this experience reveal about how our health system functions?” This reframing emphasizes the epistemic value of patient experience rather than its role as a consumer metric.

This distinction is important because experiences described as medical gaslighting are often cumulative. A single respectful interaction may occur within a larger system that repeatedly produces barriers. Institutional learning therefore requires identifying patterns rather than focusing only on isolated events. Emerging empirical work on diagnostic delay, care fragmentation, and credibility deficits supports the importance of examining longitudinal patterns of patient experience [2,3,8].

Improvement science similarly emphasizes that healthcare transformation depends on understanding systems, testing changes, and learning from performance rather than assigning responsibility solely to individuals [26]. Dixon-Woods further emphasizes that successful healthcare improvement requires attention to the complexities of organizations, implementation environments, and the unintended consequences of interventions [18]. Integrating patient experience into these learning processes may strengthen system responsiveness although empirical evaluation is still needed.

## 7. Dialogue and Shared Knowledge

Paulo Freire’s work on dialogue provides another foundation for participatory transformation [20]. Freire challenged educational models in which experts transmit knowledge to passive recipients and instead emphasized relationships based on mutual learning, inquiry, and shared authority. His dialogical framework has increasingly been applied in healthcare to support shared decisionmaking, coproduction, and collaborative problem solving [27,28,29].

This perspective has direct relevance to healthcare. Professional expertise remains essential, but healthcare decisions are strengthened when multiple forms of knowledge are recognized. Clinicians contribute biomedical knowledge while patients contribute experiential knowledge regarding symptoms, function, priorities, and illness trajectories. Healthcare organizations contribute operational knowledge regarding systems and processes. Recognizing these complementary forms of expertise aligns with recent work on relational autonomy and collaborative clinical reasoning [28,29].

A dialogical approach does not diminish professional expertise. Rather, it recognizes that complex healthcare challenges cannot be fully understood through a single perspective. Dialogue therefore becomes more than a communication technique. It becomes a mechanism for distributing knowledge and creating conditions in which healthcare systems can learn from those they serve. This approach is particularly relevant in urban healthcare environments where diverse populations and complex system interfaces require shared interpretation and co-constructed understanding [6].

## 8. Communities of Practice and Organizational Learning

Participatory knowledge production also aligns with theories of situated learning and communities of practice. Lave and Wenger proposed that knowledge develops through participation with social contexts rather than through isolated acquisition of information [30]. This perspective has been increasingly applied in healthcare to understand how professional learning, patient engagement, and organizational adaptation emerge through shared practice [31].

Healthcare organizations can therefore be understood as communities of practice in which clinicians, patients, administrators, and communities collectively develop knowledge through interaction. From this perspective, patients are not external observers of healthcare systems; they are participants whose experiences contribute to understanding and improving those systems. This reframing aligns with contemporary work on learning health systems, which emphasizes distributed knowledge creation across stakeholders [22].

This approach has important implications for medical gaslighting. If invalidation occurs, partly because patient knowledge is separated from institutional learning processes, then integrating patients into communities of practice creates opportunities for recognition, adaptation, and improvement. Studies of coproduction and collaborative governance support this claim, demonstrating that shared participation improves trust, relevance, and system responsiveness [32,33].

## 9. Translating the Framework into Urban Healthcare Practice

The proposed framework suggests that addressing medical gaslighting requires interventions across ecological levels. Because these experiences emerge through interactions among individuals, organizations, healthcare systems, and communities, solutions must extend beyond clinician communication training (Table 1).

At the clinical level, healthcare systems should support practices that recognize patients as partners in knowledge creation. These include shared decision-making, narrative-based approaches, structured opportunities for patients to describe concerns, transparent communication regarding uncertainty, and clinical processes that acknowledge experiential knowledge. Evidence-based models such as shared decision-making and narrative medicine provide practical pathways for strengthening recognition [29,34].

At the organizational level, healthcare institutions must redesign structures that limit relational care. Priorities include strengthening continuity, reducing unnecessary administrative burden, improving coordination for complex patients, and developing quality measures that assess trust, recognition, and relationship-centered care. These priorities align with improvement science frameworks emphasizing workflow redesign, team-based care, and relational coordination [35,36].

At the health system level, organizations should strengthen mechanisms that preserve patient narratives across transitions of care. Integrated information systems, patient navigation, and coordinated care models can reduce the burden placed on individuals to repeatedly reconstruct their healthcare histories. Research on shared transitions and navigation programs demonstrates improved continuity and reduced patient burden [37,38].

At the community and policy levels, healthcare systems should establish durable partnerships with communities. Participatory governance structures can help identify priorities, evaluate interventions, and ensure that improvement efforts reflect local needs [28].

Health-literate healthcare organizations similarly recognize that effective care requires systems capable of communicating clearly, reducing barriers, and responding to the needs of diverse populations [29,32]. This principle reinforces the broader argument that healthcare systems must adapt to communities rather than requiring communities to adapt exclusively to healthcare institutions.

**Figure 3:**
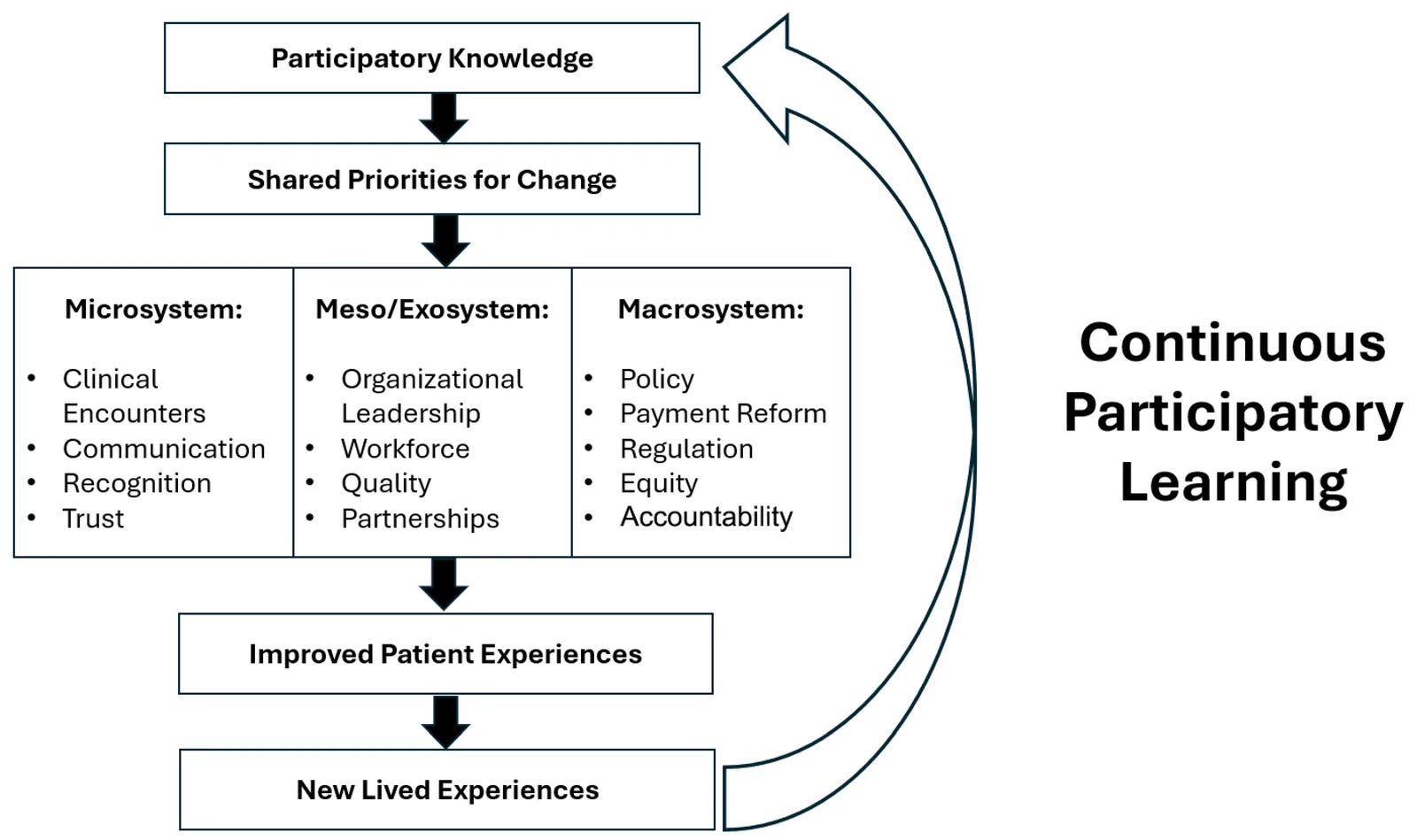
Participatory implementation model for urban healthcare transformation. Conceptual implementation framework illustrating how co-produced knowledge informs sustained healthcare system change. Community and patient priorities guide interventions across clinical, organizational, and policy domains. Clinical interventions include shared decision-making, and recognition of experiential knowledge. Organizational interventions include workflow redesign, continuity improvement, workforce development, and improvement science approaches. Policy interventions include financing alignment, accountability structures, and community partnerships. Continuous feedback mechanisms support ongoing participatory learning and adaptation [42,43,44,45,46].

Together these approaches create an implementation pathway in which patients and community experience become actionable knowledge, actionable knowledge becomes organizational learning, and organizational learning becomes sustained healthcare transformation (Figure 3). This iterative cycle reflects learning health system principles and participatory improvement models [39,40,41].

## 10. Limitations and Future Directions

This paper presents a conceptual framework and should be interpreted within that context. Although medical gaslighting has gained increasing recognition through patient narratives, public discourse, and emerging scholarly discussion, it remains an evolving construct without a universally accepted definition. Future research should develop and validate measures that examine experiences of recognition, credibility, dismissal, and trust while maintaining an appropriate distinction between harmful invalidation and appropriate clinical disagreement. Recent efforts to measure epistemic injustice and credibility deficits provide useful starting points [1,3].

The proposed bioecological framework requires empirical evaluation across ecological levels. Future studies should examine how organizational structures, workflow design, administrative burden, care continuity, financing models, and health system fragmentation influence patients’ experiences of recognition and trust. Research should also investigate how these factors interact with social determinants of health, structural inequities, and community level relationships with healthcare institutions. Mixed methods and participatory research designs may be particularly well suited for evaluating these multi-level dynamics [32].

Longitudinal research is particularly important because medical gaslighting experiences are often cumulative rather than isolated. Studies should examine how repeated healthcare interactions influence trust, healthcare utilization, disclosure of symptoms, engagement with treatment recommendations, and long-term healthcare outcomes. Understanding these temporal relationships may clarify how individual encounters contribute to broader patterns of institutional trust or distrust. Longitudinal cohort studies and repeated-measures designs could help capture these cumulative effects [1,8].

Because this framework identifies patient experience as a source of organizational learning, future research should evaluate participatory approaches as system-level interventions. Important areas of investigation include validated measures of epistemic trust in healthcare; relationships between continuity, recognition, and outcomes; organizational factors associated with reduced experiences of dismissal; community-driven approaches to healthcare improvement; and governance structures that meaningfully incorporate patients and communities into decision-making. These research priorities align with emerging work on coproduction, participatory governance, and learning health systems [31,33,39].

This framework also does not suggest that all negative healthcare experiences represent medical gaslighting. Clinical medicine necessarily involves uncertainty, interpretation, competing evidence and difficult decisions. The objective is not to eliminate disagreement between clinicians and patients, but to ensure that disagreement occurs within relationships characterized by transparency, respect, shared reasoning, and recognition of patient knowledge. Clarifying this distinction will be essential for future empirical work and conceptual refinement.

Future research should therefore focus not only on identifying when invalidation occurs, but also on understanding how healthcare systems can develop the capacity to recognize, learn, and adapt in response to patient and community experience. Developing such adaptive capacity is central to equitable, trustworthy, and human-centered care.

## 11. Conclusions

Medical gaslighting represents an important challenge for urban healthcare systems because it highlights how patients experience recognition, credibility, and trust within increasingly complex healthcare environments. Recent empirical work underscores that these experiences have shaped engagement, disclosure, and long-term relationships with healthcare institutions [1,8].

A bioecological perspective demonstrates that these experiences do not arise solely from individual interactions. Instead, they emerge through interactions among clinical relationships, organizational structures, healthcare networks, policies, social conditions, and accumulated experiences over time. This multi-level framing clarifies that medical gaslighting reflects system dynamics rather than isolated interpersonal failures.

This perspective reframes medical gaslighting from an interpersonal communication problem into an indicator of how healthcare systems manage knowledge, uncertainty, continuity and relationships. Although individual clinicians play an important role in creating respectful and trustworthy encounters, healthcare organizations also shape the conditions under which recognition and partnership are possible. Organizational constraints such as time pressure, care fragmentation, and documentation burden have been shown to influence these conditions [9,23].

Addressing medical gaslighting therefore requires more than improving communication skills. It requires healthcare systems capable of recognizing patient experience as legitimate knowledge and incorporating communities as partners in defining problems, generating solutions, and evaluating improvement. Participatory approaches, including co-design, CBPR, and experience-based coproduction, offer promising pathways for strengthening recognition and institutional learning [21,33].

Participatory knowledge production provides a pathway toward this transformation by shifting patients and communities from recipients of healthcare services to contributors in healthcare learning. Through shared inquiry, coproduction, and sustained partnership, healthcare systems can transform individual experiences into institutional knowledge and institutional knowledge into meaningful change. This iterative learning cycle aligns with learning health system principles emphasizing continuous feedback and adaptation [22].

Urban healthcare systems are uniquely positioned to benefit from this approach because the communities they serve possess essential knowledge regarding barriers, inequities, institutional relationships and opportunities for improvement. Equitable healthcare depends not only on delivering care to communities but also on building systems that learn with them. Urban environments, characterized by diversity and structural complexity, offer particularly rich contexts for participatory system transformations.

Ultimately, reducing medical gaslighting requires a transformation in how healthcare systems understand knowledge itself. The future of trustworthy healthcare depends on recognizing that expertise exists not only within institutions but also within the lived experiences of the people those institutions serve. Because this framework has not yet undergone empirical validation, its conclusions should be interpreted as theoretical propositions intended to guide future research and system design.

## Figures and Tables

**Table 1:** Ecological Levels, Mechanisms, and Participatory Interventions for Medical Gaslighting in Urban Healthcare.

Ecological Level	Theoretical Anchor	Key Breakdown (What Is Not Working)	Manifestation in Urban Healthcare	Policy and Practice Solutions
**Microsystem** Patient Clinician Interaction	Miranda Fricker Epistemic Injustice	Asymmetric epistemic authority Credibility deficits Epistemic conflict Structural epistemic invalidation Patient knowledge undervalued	Symptom dismissal Psychologizing Premature attribution Disbelief of patient narratives	Narrative-based clinician training Structured patient narrative time Shared decision-making tools Integrated patient reported outcomes Communication standards validating the patient’s experience
**Mesosystem** Healthcare Organizations	Michael Lipsky Street-level Bureaucracy	Time scarcity Productivity pressures Workflow rigidity Competing institutional demands Reliance on heuristics Burnout	Rushed visits Premature diagnostic closure Deflection Reduced listening and relational care Limited follow-up	Complexity-adjusted scheduling Reform productivity metrics to include trust Team-based care Case reviews for unresolved symptoms Reduced documentation burden Trust-centered quality measures
**Exosystem** Health System Structures	Health Systems Theory	Fragmented care Discontinuity Administrative/Referral barriers Siloed specialties	Repeated storytelling across providers Inconsistent diagnoses Loss to follow-up Navigation burdens	Integrated care coordination and records Patient navigation programs Transparent referral tracking Incentives for continuity Shared patient narratives
**Macrosystem** Policy and Structural Environment	Structural Inequality and Political Economy of Healthcare	Insurance/Financing constraints Fee-for-service Reimbursement Incentives Limited coverage Administrative burdens Systemic inequities Unequal resource distribution	Unequal access Time limited visits Restricted/Delayed diagnostics Efficiency prioritized over relational care	Payment reform: value-based, relational care Expanded coverage for complex/uncertain conditions Administrative burden simplification Equity focused accountability Fund Community health initiatives
**Chronosystem** Time and Accumulated Experience	Bronfenbrenner Life Course	Repeated invalidation and erosion of trust over time Repeated dismissal Diagnostic delays Distrust Doubt	Care avoidance Delayed presentation Community distrust	Longitudinal trust/experience tracking Incent long-term patient continuity Escalate early for persistent symptoms Community-based participatory trust building
**Cross-Cutting Transformation**	Dewey, Freire, and Community-Based Participatory Research	Limited institutional learning from patient experience	Communities viewed as recipients rather than knowledge partners	Participatory governance Co-produced interventions Community-engaged healthcare improvement

## Data Availability

No new data were created or analyzed in this study. Data sharing is not applicable to this article.
